# Prognostic role of carotid intima-media thickness in off-pump coronary artery bypass surgery

**DOI:** 10.1038/s41598-018-29863-z

**Published:** 2018-07-30

**Authors:** Sung-Yeon Ham, Jong-Wook Song, Jae-Kwang Shim, Sarah Soh, Hee-Jung Kim, Young-Lan Kwak

**Affiliations:** 1 Department of Anesthesiology and Pain Medicine, Yonsei University College of Medicine, Seoul, Republic of Korea; 2 Anesthesia and Pain Research Institute, Yonsei University College of Medicine, Seoul, Republic of Korea; 3 Yonsei Cardiovascular Hospital, Yonsei University College of Medicine, Seoul, Republic of Korea

## Abstract

Carotid intima-media thickness (IMT) is a well-known predictor of adverse outcomes in the ischemic heart disease patients; however, evidence is lacking in patients undergoing off-pump coronary artery bypass surgery (OPCAB). Data from 407 patients who underwent OPCAB between April 2013 and August 2016 were retrospectively reviewed. A composite of cardiovascular morbidity endpoints was defined as the presence of stroke, acute myocardial infarction, new cardiac arrhythmia (newly developed atrial fibrillation, atrial flutter, or atrioventricular block), cardiovascular death, or cerebrovascular death within 30 days after surgery. Increased carotid IMT was defined as ≥0.9 mm on one or both sides. The incidence of a composite of cardiovascular morbidity endpoints was 24.0% in the normal IMT group (n = 221) and 34.4% in the increased IMT group (n = 186) (*p = *0.021). Multivariable analysis revealed increased IMT (odds ratio 1.719, 95% confidence interval 1.108 to 2.666, p = 0.016) and preoperative renal replacement therapy (odds ratio 4.264, 95% confidence interval 1.679 to 10.829, p = 0.002) as independent predictors of a composite of cardiovascular morbidity endpoints. In patients undergoing OPCAB, preoperative assessment of carotid IMT may help predicting the development of a postoperative composite of cardiovascular morbidity endpoints.

## Introduction

The development of intimal thickening has been demonstrated to create a substrate for atherosclerotic stimuli, predisposing the artery to the development of advanced atherosclerosis^1,2^. In conjunction, carotid intima-media thickness (IMT) is being considered a preclinical surrogate marker reflecting atherosclerotic burden, endothelial dysfunction, and target organ damage^3–5^. Indeed, ample evidence is available supporting the role of carotid IMT as a strong predictor of cardiovascular outcomes in nonsurgical populations of either asymptomatic or diseased patients^5,6^. Moreover, its predictive role extends to patients with ischemic heart disease undergoing percutaneous coronary intervention (PCI), who have advanced atherosclerosis that has progressed far beyond the preclinical stage^7^.

In addition, patients undergoing coronary artery bypass graft (CABG) surgery are at a high risk of developing adverse cardiovascular outcomes. Accordingly, risk prediction models utilizing traditional risk factors have been proposed by responsible societies in an effort to improve risk evaluation and outcome^8–10^. However, the accuracies of the two most widely used risk models (European System for Cardiac Operative Risk Evaluation [EuroSCORE] and the Society of Thoracic Surgeons Risk Score) are subject to debate^8,10–12^, and they do not incorporate parameters for directly assessing the patient’s vascular status, such as IMT. Yet, in contrast to the encouraging results in the PCI setting^7^, the prognostic value of carotid IMT could not be observed in patients undergoing on-pump CABG^13^.

Off-pump CABG (OPCAB) serves as an important technique for surgical revascularization, which may be preferred in patients with cerebrovascular risk factors^14^ and in elderly patients^15^. Theoretically, it imposes the additional burden of surgery on coronary artery disease and PCI, yet avoids risks related to cardiopulmonary bypass (CPB). Therefore, the predictive role of carotid IMT on a composite of cardiovascular morbidity endpoints after OPCAB may be different from those after PCI or on-pump CABG; however, this has not been addressed before.

In this retrospective study, we tested our hypothesis that preoperatively evaluated carotid IMT could be a meaningful predictor of a composite of cardiovascular morbidity endpoints after OPCAB.

## Materials and Methods

**Figure 1:**
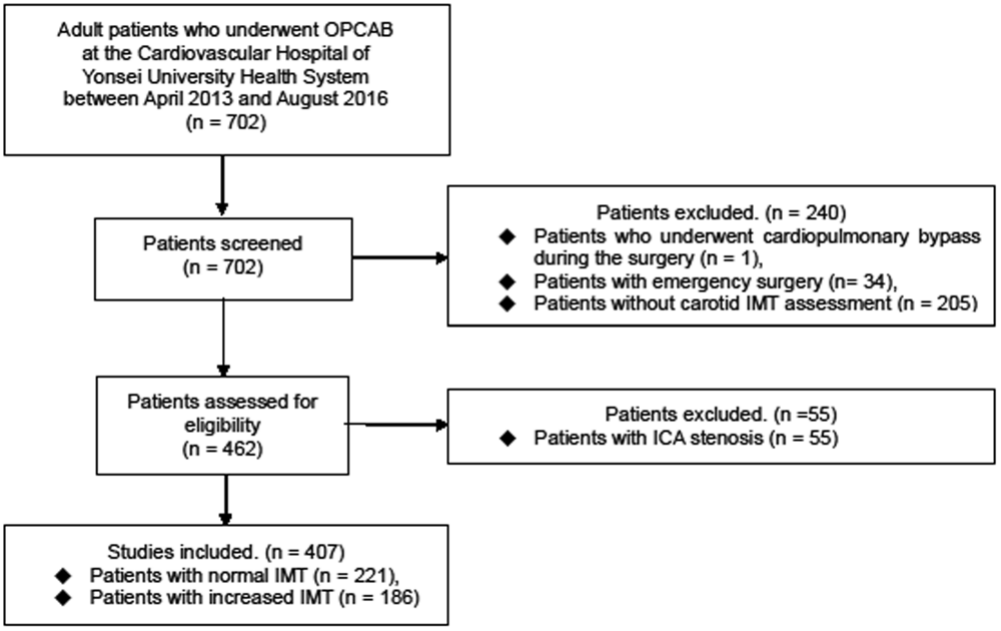
Study flowchart. ICA, internal carotid artery; IMT, intima-media thickness; OPCAB, off-pump coronary bypass surgery.

After receiving approval from the Institutional Review Board of Severance Hospital (4-2015-1002), we retrospectively reviewed the medical records of adult patients who underwent isolated OPCAB at the Cardiovascular Hospital of Yonsei University Health System between April 2013 and August 2016. The need for informed consent was waived. During the study period, a total of 702 consecutive patients underwent OPCAB. Patients who underwent cardiopulmonary bypass during the surgery (n = 1), emergency surgery (n = 34), and without carotid IMT assessment within 1 month before surgery (n = 205) were excluded. Among patients assessed with carotid IMT, patients with internal carotid artery stenosis >50% were also excluded (n = 55) due to the lack of feasibility to measure carotid IMT accurately and well-known high risk of postoperative neurologic complications. A total of 407 patients were enrolled in the current study, and divided into the normal IMT group (n = 221) and increased IMT group (n = 186) (Fig. 1). Increased carotid IMT was defined as carotid IMT ≥ 0.9 mm on one or both sides.

### Study end points

The primary end point was to compare the incidence of a 30-day composite of cardiovascular morbidity endpoints after OPCAB between the groups and to investigate whether increased carotid IMT is an independent risk factor for developing a composite of cardiovascular morbidity endpoints. A composite of cardiovascular morbidity endpoints was defined as the presence of stroke, acute myocardial infarction (creatinine kinase-MB > 25 ng/mL, newly developed Q wave on electrocardiography, or development of left bundle branch block), new cardiac arrhythmia (newly developed postoperative atrial fibrillation, atrial flutter, or atrioventricular block), or cardiovascular or cerebrovascular death within 30 days after OPCAB. Based on the prognostic importance of postoperative new cardiac arrhythmia in cardiac surgical patients^16,17^ and the close relationship between the increased carotid IMT and the occurrence of atrial fibrillation in medical patients^18–20^, we included new cardiac arrhythmia in the composite of cardiovascular morbidity end points.

### Carotid ultrasonography

The carotid IMT was assessed with high-resolution B-mode ultrasonography, with an 11-MHz imaging transducer (Vivid-7, Vivid E9 [GE, Milwaukee, WI, USA], SC2000 [Siemens, Mountain View, CA, USA], and Sonos 7500 or iE33 [Philips, Andover, MA, USA]). Ultrasonographic images of the carotid artery were acquired in end-diastolic phase. Carotid IMT was measured at the far vessel wall at a site approximately 1 cm proximal to the carotid bulb. All ultrasonographic measurements were performed by sonographers certified by the Registry of Diagnostic Cardiac Sonographers.

### Preoperative measurements

The investigated baseline patient characteristics were sex; age; body surface area; history of diabetes, hypertension, cerebrovascular accident, and chronic kidney disease; left ventricular ejection fraction; extent of coronary artery disease; and EuroSCORE II^8,10^.

### Intraoperative management and measurement

Standardized anesthetic care according to an institutional protocol was provided to all patients. On transfer to the operating room, five-lead electrocardiography, pulse oximetry, and invasive arterial blood pressure monitoring were conducted. Anesthesia was induced with midazolam 0.03–0.05 mg/kg and sufentanil 1.5–2 μg/kg. Tracheal intubation was facilitated with rocuronium bromide 50 mg. Patients were ventilated with 40% oxygen, tidal volume of 8 mL/kg, respiratory rate of 8–14/min, and positive end-expiratory pressure of 5 mm Hg. Anesthesia was maintained with 0.6–2.0 vol% of sevoflurane and sufentanil 0.5–1.5 μg·kg^−1^·h^−1^. Transesophageal echocardiography (TEE) was performed to evaluate cardiac function, to detect newly developed regional wall motion abnormality or mitral regurgitation, and to record the Katz grade (Grade 1 = normal to mild intimal thickening, Grade 2 = severe intimal thickening, Grade 3 = atheromas protruding <5 mm into the aortic lumen, Grade 4 = atheromas protruding ≥5 mm into the aortic lumen, Grade 5 = with mobile components)^21^. A pulmonary artery catheter was inserted to monitor cardiac output and pulmonary arterial pressure. Intraoperatively, balanced crystalloid (Plasma solution A 1000 inj; CJ, Seoul, Korea) was infused at 5–6 mL·kg^−1^·h^−1^, and balanced synthetic colloid (Volulyte; Fresenius Kabi, Bad Homburg, Germany) was used to replace estimated blood loss at a maximum dose of 1000 mL. A cell-saving device was used, and collected blood was reinfused at the end of the operation. Mean arterial pressure (MAP) was maintained at >70 mm Hg with norepinephrine infusion and 5–10° Trendelenburg position during grafting. If the norepinephrine infusion dose was >0.2 µg·kg^−1^·min^−1^ to achieve the target MAP, vasopressin (up to 4 IU/h) was added in a stepwise fashion. Milrinone (0.5 µg·kg^−1^·min^−1^) was infused if mixed venous oxygen saturation was persistently low (SvO_2_, <60% for >10 min) or severe mitral regurgitation (grade ≥ 3) was newly developed. Allogeneic packed erythrocytes (pRBCs) were transfused when the hematocrit decreased to <25% throughout the study period.

The number of grafts performed; duration of anesthesia and operation; amounts of vasopressors and inotropics; hemodynamics including heart rate, MAP, cardiac index, and mixed venous oxygen saturation (SvO_2_); amount of intraoperative fluid administration and urine output; and amount of reinfused blood via cell salvage and pRBC transfusion were recorded. All patients were transported to the intensive care unit (ICU) after the operation.

### Postoperative measurements

The amount of blood loss and transfusion within postoperative 24 hours, and the lengths of ICU and hospital stay were recorded postoperatively.

### Statistical analysis

All statistical analyses were performed with SPSS 23.0 (IBM Corp. Released 2015. IBM SPSS Statistics for Windows, Version 23.0. Armonk, NY: IBM Corp). For continuous variables, descriptive statistical values were calculated and reported as mean ± standard deviation. Categorical variables were described by using frequency distributions. Student’s t-test or the Mann-Whitney U-test were used to detect differences in the mean of continuous variables, and the chi-square test or Fisher’s exact test was used to compare categorical variables between Normal IMT group and Increased IMT group. Because age at operation differed between the groups, multiple linear regression analysis was performed. Logistic regression analysis was conducted to investigate independent risk factors for composite of cardiovascular morbidity endpoints after OPCAB. To evaluate possible risk factors for composite of cardiovascular morbidity endpoints, a univariable logistic regression analysis was performed. For the multivariable model, a stepwise selection method was used to select variables that showed *p* < 0.05 in the univariable analysis. A value of *p* < 0.05 was considered statistically significant.

## Results

During the study period, a total of 407 patients were retrospectively identified. Of them, 221 patients exhibited normal carotid IMT (<0.9 mm), whereas the remaining 186 patients exhibited increased carotid IMT (≥0.9 mm) (Fig. 1).

There were no significant differences between the groups in baseline characteristics including preoperative medications and the severity of coronary disease, except age. Patients in the increased IMT group were older than those in the normal IMT group (66.3 ± 8.4 years *vs*. 62.2 ± 9.8 years, *p* < 0.001). Katz grade (3.7 ± 0.7 *vs*. 3.4 ± 0.8, *p* < 0.001) investigated with intraoperative TEE and the incidence of Katz grade ≥ 4 (62.9% *vs*. 46.2%, *p* = 0.001) were both significantly higher in the increased IMT group than in the normal IMT group (Table 1).

**Table 1:** Demographic and perioperative clinical data.

	Normal IMT (n = 221)	Increased IMT (n = 186)	*p* value
Sex (female)	41 (18.6)	32 (17.2)	0.724
Age (years)	62.2 ± 9.8	66.3 ± 8.4	<0.001*
BSA (m^2^)	1.8 ± 0.2	1.7 ± 0.2	0.645
DM	125 (56.6)	104 (55.9)	0.896
HTN	149 (67.4)	134 (72.0)	0.313
CVA	18 (8.1)	21 (11.3)	0.283
CKD/preoperative RRT	35 (15.8)/12 (5.4)	25 (13.4)/8 (4.3)	0.497/0.600
EF (%)	55.4 ± 13.9	53.3 ± 15.3	0.158
Left main disease	49 (22.2)	52 (28.1)	0.176
Number of graft	3.4 ± 0.8	3.3 ± 0.8	0.800
Number of diseased vessels	2.9 ± 0.4	2.9 ± 0.3	0.989
1 vessel disease	1 (0.5)	0	1.000
2 vessel disease	29 (13.1)	26 (14.0)	0.884
3 vessel disease	191 (86.4)	160 (86.0)	1.000
Unstable angina	126 (57.0)	98 (52.7)	0.355
MI within 1 week	39 (17.7)	35 (18.8)	0.777
NYHA III or IV	24 (10.9)	28 (15.1)	0.221
EuroSCORE II	4.1 ± 2.8	4.7 ± 2.7	0.051
Beta blocker	119 (53.8)	92 (49.5)	0.426
RAS antagonists	96 (43.4)	92 (49.5)	0.233
Statin	169 (76.5)	141 (75.8)	0.907
Katz grade/grade ≥ 4	3.4 ± 0.8/102 (46.2)	3.7 ± 0.7/117 (62.9)	<0.001*/0.001*
Preoperative CK-MB (μg/L)	2.5 ± 4.6	3.2 ± 10.8	0.405
Preoperative Cr (mg/dL)/eGFR	1.3 ± 1.8/80.8 ± 25.9	1.2 ± 1.2/79.9 ± 23.2	0.208/0.691

Values are presented as the mean ± standard deviation or number of patients (%). *p < 0.05.

BSA, body surface area; CKD, chronic kidney disease; CK-MB, serum creatinine kinase-MB level; Cr, serum creatinine level; CVA, cerebrovascular accident; DM, diabetes mellitus; EF, ejection fraction; eGFR (mL·min^−1^·1.73 m^−2^), estimated glomerular filtration rate; EuroSCORE II, European System for Cardiac Operative Risk Evaluation II; HTN, hypertension; left main disease, left main coronary artery stenosis >50%; MI, myocardial infarction; NYHA, New York Heart Association Functional Classification; RAS, renin-angiotensin system; RRT, renal replacement therapy.

The incidence of a 30-day composite of cardiovascular morbidity endpoints (34.4% *vs*. 24.0%, *p* = 0.021) was significantly greater in the increased IMT group than in the normal IMT group. The incidence of 30-day all-cause mortality (normal IMT; 1.4% *vs*. increased IMT; 2.7%, *p* = 0.478), and the lengths of ICU and hospital stay and were similar between the groups (Table 2).

**Table 2:** Postoperative outcomes.

	Normal IMT (n = 221)	Increased IMT (n = 186)	*p* value
30 d composite of cardiovascular morbidity endpoints	53 (24.0%)	64 (34.4%)	0.021*
Postop stroke	2 (0.9%)	4 (2.2%)	0.419
Postop MI	8 (3.6%)	9 (4.8%)	0.540
Postop arrhythmia	41 (18.6%)	47 (25.3%)	0.101
Cardiovascular mortality	2 (0.9%)	4 (2.2%)	0.419
Cerebrovascular mortality	0	0	N/A
30 d-all cause mortality	3 (1.4%)	5 (2.7%)	0.478
ICU day (day)	2.9 ± 3.1	2.9 ± 2.6	0.991
Hospital day (day)	14.4 ± 12.3	14.1 ± 8.5	0.773

Values are presented as the mean ± standard deviation or number of patients (%). *p < 0.05.

MI, myocardial infarction; Postop arrhythmia, newly developed postoperative atrial fibrillation, atrial flutter, or atrioventricular block; 30 d-all cause mortality, mortality with any cause within postoperative 30 days; ICU, intensive care unit.

Intraoperative data including the number of grafts performed, fluid balance, and transfusion requirement were similar between the groups. The total amount of norepinephrine infused during the operation was significantly greater in the increased IMT group than in the normal IMT group (909.0 ± 598.9 μg *vs*. 736.2 ± 543.8 μg, *p* = 0.029). The number of patients who required vasopressin was also significantly greater in the increased IMT group than in the normal IMT group (57.7% *vs*. 44.6%, *p = *0.017). Intraoperative hemodynamics (data not shown) including the lowest cardiac index and SvO_2_ were similar between the groups (Table 3).

**Table 3:** Operation related data.

	Normal IMT (n = 221)	Increased IMT (n = 186)	*p* value
Graft number	3.4 ± 0.8	3.3 ± 0.8	0.800
Anesthesia time (min)	304.7 ± 39.5	309.8 ± 38.6	0.190
Operative time (min)	232.4 ± 39.4	237.9 ± 47.5	0.198
Total ischemic time (min)	36.5 ± 9.3	35.6 ± 8.3	0.366
Amount of crystalloid (mL)	1698.2 ± 642.3	1764.1 ± 543.9	0.279
Amount of colloid (ml)	478.4 ± 244.5	492.4 ± 201.4	0.538
Urine output (ml)	300.2 ± 224.2	338.5 ± 272.4	0.127
pRBC transfusion (ml)	64.5 ± 161.9	65.8 ± 144.8	0.928
Cell saver (ml)	253.7 ± 149.3	251.5 ± 134.5	0.883
Patients with norepinephrine	178 (99.4%)	152 (99.3%)	1.000
Total norepinephrine (mcg)	736.2 ± 543.8	909.0 ± 598.9	0.029*
Patients with vasopressin	79 (44.6%)	90 (57.7%)	0.017*
Patients with milrinone	23 (13.0%)	21 (13.5%)	1.000
Lowest cardiac index (L/m^2^)	1.6 ± 0.3	1.7 ± 0.4	0.367
Lowest SvO_2_ (%)	70.6 ± 7.8	69.8 ± 7.6	0.586
Bleeding for postop 24 h (ml)	681.2 ± 462.4	646.5 ± 373.0	0.407
pRBC transfusion for postop 24 hrs (ml)	112.8 ± 224.9	88.8 ± 181.5	0.240

Values are presented as the mean ± standard deviation or number of patients (%).

Cell saver, amount of reinfused blood by the cell salvage technique; SvO_2_, mixed venous oxygen saturation; pRBC, packed red blood cell.

Among the variables assessed in the univariable analysis, increased IMT, age, and preoperative renal replacement therapy (RRT) showed *p* < 0.05, and were further introduced into multivariable analysis. In multivariable logistic regression analysis to detect independent factors of a composite of cardiovascular morbidity endpoints after OPCAB, increased IMT (odds ratio 1.719, 95% confidence interval 1.108 to 2.666, p = 0.016) and preoperative RRT (odds ratio 4.264, 95% confidence interval 1.679 to 10.829, p = 0.002) remained as independent risk factors for 30-day composite of cardiovascular morbidity endpoints (Table 4).

**Table 4:** Logistic regression analysis for predictors of 30-day composite of cardiovascular morbidity endpoints.

	Univariable		Multivariable	
	OR (CI)	*p* value	OR (CI)	*p* value
Increased IMT	1.741 (1.081–2.807)	0.023*	1.719 (1.108–2.666)	0.016*
Age	1.012 (0.983–1.043)	0.423		
DM	0.777 (0.486–1.243)	0.292		
HTN	1.372 (0.793–2.374)	0.259		
CVA	1.304 (0.613–2.774)	0.491		
Preoperative RRT	4.178 (1.466–11.911)	0.007*	4.264 (1.679–10.829)	0.002*
Recent MI	0.941 (0.497–1.782)	0.851		
Katz grade ≥ 4	1.077 (0.650–1.783)	0.774		
EuroSCORE II	1.012 (0.918–1.116)	0.811		

Values are presented as the odds ratio (95% confidential interval). *p < 0.05.

Increased IMT, carotid intima-media thickness ≥0.9 mm on one or both sides; DM, diabetes mellitus; HTN, hypertension; CVA, cerebrovascular accident; RRT, renal replacement therapy; Recent MI, myocardial infarction within 1 wk; EuroSCORE II, European System for Cardiac Operative Risk Evaluation II.

## Discussion

In this retrospective study addressing the impact of preoperative carotid IMT on postoperative outcome in OPCAB, we observed a significantly more frequent occurrence of a 30-day composite of cardiovascular morbidity endpoints in the increased IMT (≥0.9 mm) group than in the normal IMT group. Moreover, only increased carotid IMT was revealed to be independently associated with a 1.7-fold increase in the risk of developing a 30-day composite of cardiovascular morbidity endpoints along with preoperative requirement for RRT, which showed a 4.3-fold increased risk.

In the management of patients undergoing cardiac surgery, the importance of risk scores for identifying high-risk patients is widely appreciated not only for accurate risk stratification but also for the application of potentially beneficial preventive strategies in order to improve prognosis. Currently, most of the validated risk scoring systems are based on combinations of traditional risk factors that mainly reflect the preoperative co-morbid disease status and functional capacity of the patients, and factors related to the specific risk inherent to the type of surgery^11,12^. With particular attention to patients undergoing CABG surgery, distinct efforts have been made to predict postoperative morbidity more accurately by including the vascular status of the patient. Among them, the presence of protruding atheroma investigated with TEE has been shown to be an important risk factor for perioperative stroke in patients undergoing CABG^22^. TEE is an invaluable monitoring tool during CABG; however, it is a relatively invasive method and not routinely performed preoperatively in patients requiring isolated CABG. Thus, a noninvasive, simple, and preoperatively feasible method such as carotid ultrasonography has been proposed as a screening test for cardiovascular events.

Among parameters measured with carotid ultrasonography, IMT measured with B-mode ultrasound reflects the process of arterial wall change in association with atherosclerosis as a continuous variable^1^. Carotid IMT has been used as an indicator of generalized vascular atherosclerosis and arterial aging^3^, as well as an index to predict the degree of atherosclerosis of the coronary arteries^23^. In medical patients, increase in carotid IMT was closely associated with increases of the relative risks for stroke and myocardial infarction in long-term follow-up studies^5,6,24,25^, and was a strong predictor of cardiovascular events^5^, even in patients without known cardiovascular disease^26^. Recent studies also revealed that increased carotid IMT can also predict the occurrence of atrial fibrillation in various medical cohorts^19,20,27^. This association was suggested to be attributable to atrial hypoperfusion and ischemia caused by arterial structural change^28^, while postoperative arrhythmia is a well-known harbinger of adverse outcome, especially after CABG^16^. These findings proved the importance of carotid IMT as a surrogate end point for atherosclerosis and target organ damage^3^. Furthermore, in a previous study, decrease in carotid IMT with treatment strategies resulted in the reduction of vascular events^29^, indicating that carotid IMT would be a validated marker for the reversibility of atherosclerosis and serve as a guide for therapies^3^. Despite its solid background in medical patients, only a dearth of researches have evaluated the validity of carotid IMT as a predictor of postoperative cardiovascular outcome in patients undergoing CABG.

Notably, by avoiding CPB, OPCAB should possess distinct characteristic features of reduced systemic atherosclerotic embolic burden^30^. On the other hand, inevitable hemodynamic derangements and reduction in cardiac output during distal anastomosis may elicit organ damage, especially when endothelial dysfunction is present. Moreover, compliance of the vascular system assessed at the carotid artery has been shown to be closely related to the degree of hemodynamic compromise during grafting in OPCAB, possibly owing to vascular-ventricular coupling^31^. Therefore, preoperative vascular status as assessed by using carotid IMT would have clinical relevance in predicting a composite of cardiovascular morbidity endpoints after OPCAB, which has not been validated before. The reported benefits and related preference for OPCAB in elderly patients^15^ and in certain groups of high-risk patients for cerebrovascular events^32^ also add to the importance of vascular status as a predictor of a composite of cardiovascular morbidity endpoints after OPCAB.

As our results indicate, increased IMT of ≥0.9 mm at any one of the carotid arteries was an independent prognostic factor for a 30-day composite of cardiovascular morbidity endpoints after OPCAB along with preoperative RRT, whereas the EuroSCORE II was similar between the normal IMT and increased IMT groups. This finding is in accordance with the previous result in patients undergoing vascular surgery that delineated prognostic values of increased carotid IMT to predict cardiovascular events and mortality^33^. In patients with coronary artery disease, the prognostic value of increased carotid IMT for cardiovascular events could be proven in patients undergoing PCI^7^; however, a study conducted in patients undergoing on-pump CABG could not prove it^13^, contrary to the results of the current study. This discrepancy might be primarily associated with the use of CPB. By excluding the impact of systemic embolic atheromatous burden by large aortic cannulas, mechanically driven flow, and cross clamps^34^, increased carotid IMT, as a reflection of the indwelling vascular status itself, may have played a more decisive role on the occurrence of a composite of cardiovascular morbidity endpoints after OPCAB, which is consistent with the PCI setting^7^. In that context, Katz grade, a definite risk factor for cardiovascular events in on-pump CABG, was not an independent risk factor for a composite of cardiovascular morbidity endpoints in the present study, although the number of patients with Katz grade ≥4 were significantly greater in the increased IMT group. Since the incidence of adverse outcome following coronary revascularization surgery was demonstrated to be different between OPCAB and on-pump CABG^15,32^ and possible etiologies might be different due to factors including the use of CPB, the risk stratification method should be individualized according to the method of the surgery. In this regard, the strength of this study was to examine the risk assessment tool in patients with OPCAB who differ from on-pump CABG patients.

We also observed a significantly higher vasopressor requirement in the increased IMT group than in the normal IMT group during the operation in order to maintain the preset target MAP. This might also be attributed to reduced vascular compliance^31^ and increased blood pressure variability^35,36^, which are characteristic features of patients with increased carotid IMT. Increased blood pressure variability was reported to markedly contribute to the increased risk of cardiovascular events in nonsurgical patients with increased carotid IMT, supporting the close relationship between the vasopressor requirement and carotid IMT.

The finding that age, a previously well-known risk factor of adverse outcome after OPCAB^10^, could no longer be identified as an independent risk factor of a composite of cardiovascular morbidity endpoints after multivariable analysis may be attributable to its close relationship with carotid IMT^37^. Accordingly, our results also show that patients in the increased IMT group were significantly older than those in the normal IMT group. Thus, the interdependence of these variables may have caused the exclusion of age as a risk factor, whereas increased carotid IMT more directly reflects the atherosclerotic burden, endothelial dysfunction, and target organ damage than age per se^3–5^.

Of interest, 54% of the studied patients exhibited normal carotid IMT (<0.9 mm) despite having advanced atherosclerosis of the coronary artery requiring surgical revascularization. This observation verifies that increased carotid IMT is not merely a part of the preclinical disease process of atherosclerosis but also subject to modulation by various stimuli on the carotid artery^3^. It also implies that carotid IMT has a prognostic significance beyond that of a surrogate marker of atheroma burden. Whether incorporation of carotid IMT to established risk prediction models would improve their accuracies on predicting outcome merits a further large-scale study.

This study has several limitations. First, this study is subject to limitations inherent to its retrospective nature. Second, we excluded patients who presented as emergency cases and those with carotid artery stenosis, as carotid IMT measurement was not feasible in these patients. Apart from the inability to measure the IMT, ICA stenosis is also an obvious risk factor of postoperative neurologic complications^38^, that would not fit the purpose of the current study. Third, we used a cutoff value of ≥0.9 mm, which was validated by a vast amount of literature^39^. However, various IMT values (≥1.11 mm, ≥1.25 mm) for the prediction of cardiovascular or cerebrovascular complications were used in the above-cited studies, which cannot be addressed in the current study without seriously sacrificing the statistical power. Another limitation is a relatively small sample size, which was not enough to investigate the association of IMT with individual cardiovascular events. Thus, only a composite of cardiovascular morbidity endpoints was compared between the two groups. Lastly, only short-term follow up records were included in the current study. Further studies regarding long-term follow up results will be needed.

## Conclusion

In conclusion, this retrospective analysis firstly reveals that increased carotid IMT (≥0.9 mm) was independently associated with a 1.7-fold increased risk of a 30-day composite of cardiovascular morbidity endpoints in patients undergoing OPCAB, along with the preoperative requirement for RRT. Our results provide evidence suggesting that routine assessment of preoperative carotid IMT may allow a more comprehensive risk stratification in patients undergoing OPCAB.

### Data availability statement

Materials, data and associated protocols are available.
